# Supplementary material for: Methyl transfer in psilocybin biosynthesis
Source: Nat Commun. 2024 Mar 28;15:2709. doi: 10.1038/s41467-024-46997-z (PMC10978996; doi:10.1038/s41467-024-46997-z)
Supplement: Supplementary file 1 — Supplementary Information [file 41467_2024_46997_MOESM1_ESM.pdf]

Supplementary Fig. 1

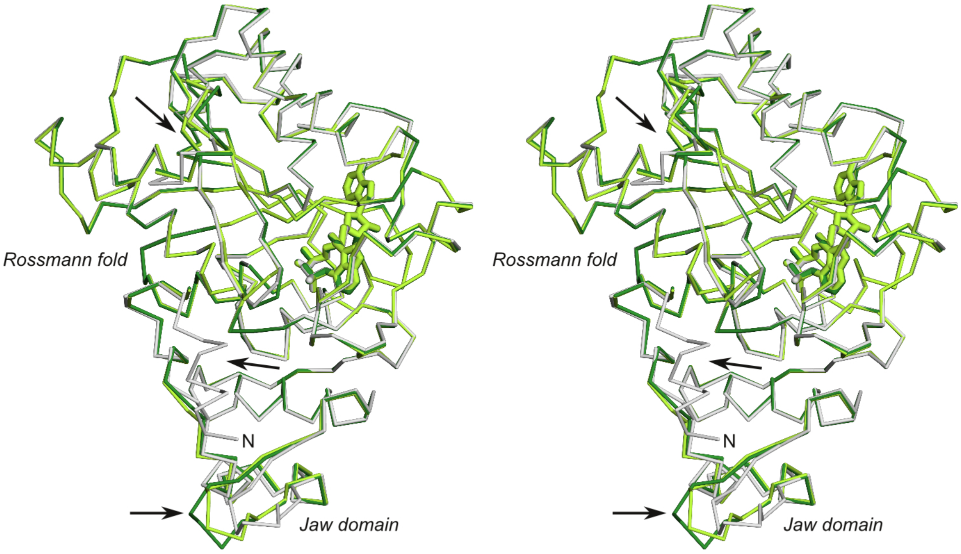

Comparison of the orthorhombic and monoclinic crystal forms of the PsiM-SAH-norbaeocystin complex.

Wall-eyed stereo view showing the A and B chains of the monoclinic crystal form (PDB entry 8PB3, chains A and B coloured bright and dark green, respectively) superposed onto the orthorhombic model (PDB entry 8PB4, grey). Protein chains are shown as C_α-traces, SAH and norbaeocystin (main conformation only) as stick models. The two protein chains in the ASU of the monoclinic form are virtually identical and can be superimposed onto one another with a C_α-RMSD of 0.20 Å (310 superimposed atoms) and onto the orthorhombic structure with C_α-RMSD values of 0.63 Å (chain A, 310 superimposed atoms) and 0.62 Å (chain B, 310 superimposed atoms). Arrows indicate areas that are different.

Supplementary Fig. 2

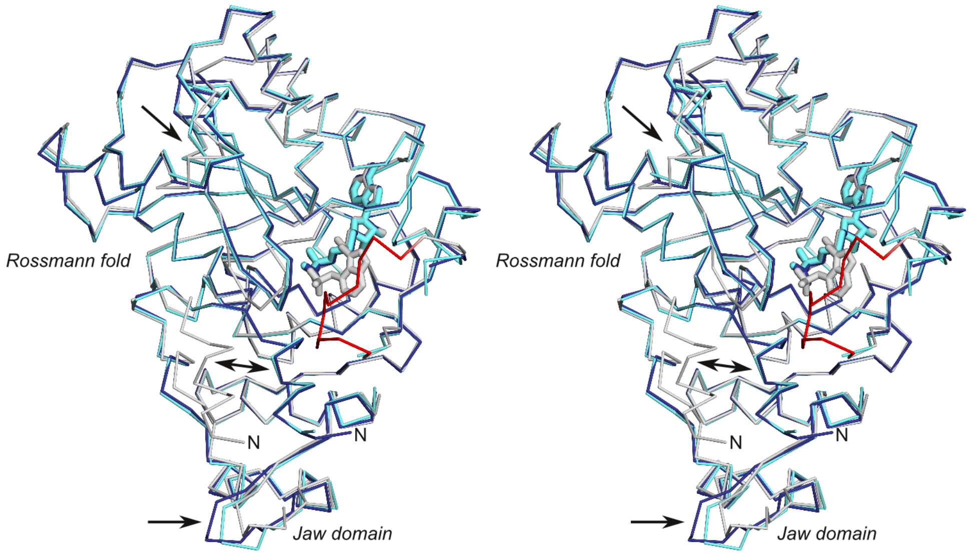

Comparison of the orthorhombic crystal form of the PsiM-SAH-norbaeocystin complex and the tetragonal PsiM-SAH complex.

Wall-eyed stereo view showing the A and B chains of the substrate-free tetragonal crystal form (PDB entry 8PB8, chains A and B coloured bright and dark blue, respectively) superposed onto the orthorhombic model (PDB entry 8PB4, grey, with the SRL region that is missing in the substrate-free structures highlighted in red). Protein chains are shown as C_α-traces, SAH and norbaeocystin (main conformation only) as stick models. The two protein chains in the ASU of the tetragonal form are highly similar and can be superimposed onto one another with a C_α-RMSD of 0.37 Å (309 superimposed atoms) and onto the orthorhombic structure with C_α-RMSD values of 0.74 Å (chain A, 299 superimposed atoms) and 0.78 Å (chain B, 297 superimposed atoms). Arrows indicate areas that are different.

Supplementary Fig. 3

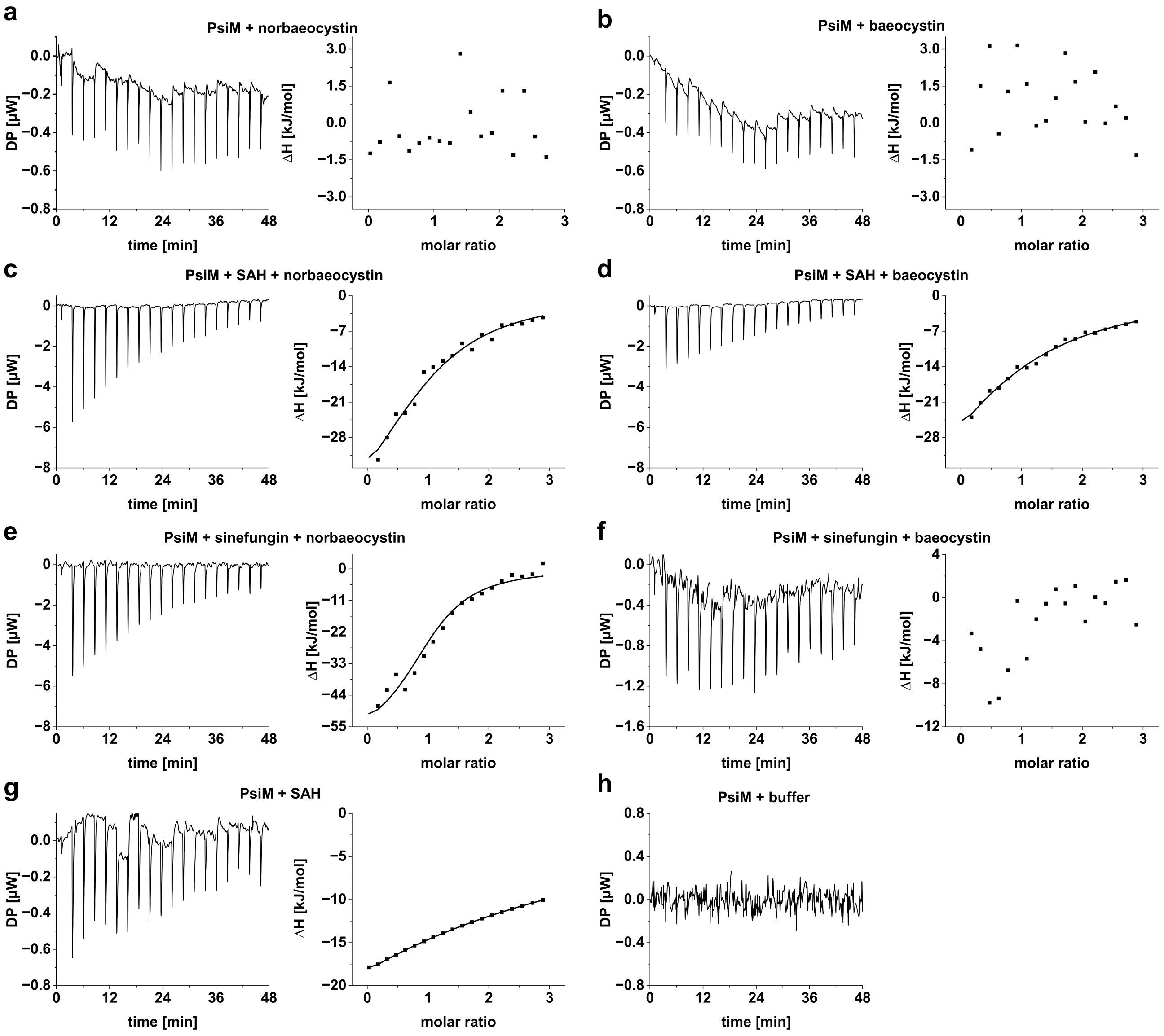

Interaction studies using isothermal titration calorimetry (ITC).

Analysis of substrate binding by PsiM in the presence and absence of a cofactor analogue (either SAH or the SAM-like inhibitor sinefungin). Left graphs within panels **a**–**h** show the differential power (DP), right panels the enthalpy (ΔH, not shown for the buffer control in panel **h**).

a, b. Titration with norbaeocystin or baeocystin in the absence of cofactor analogues.

c, d. Titration with norbaeocystin or baeocystin in the presence of SAH. Experimentally determined K_d values: 35.7 ± 5.4 µM and 77.2 ± 3.5 µM, respectively.

e, f. Titration with norbaeocystin or baeocystin in the presence of sinefungin. Experimentally

determined K_d value: 10.7 ± 0.8 μM for norbaeocystin.

g. Titration with SAH in the absence of norbaeocystin and baeocystin. Experimentally determined K_d value: 66 ± 49 μM.

h. Negative control, titration with 50 mM Tris buffer (pH 8.4).

Supplementary Fig. 4

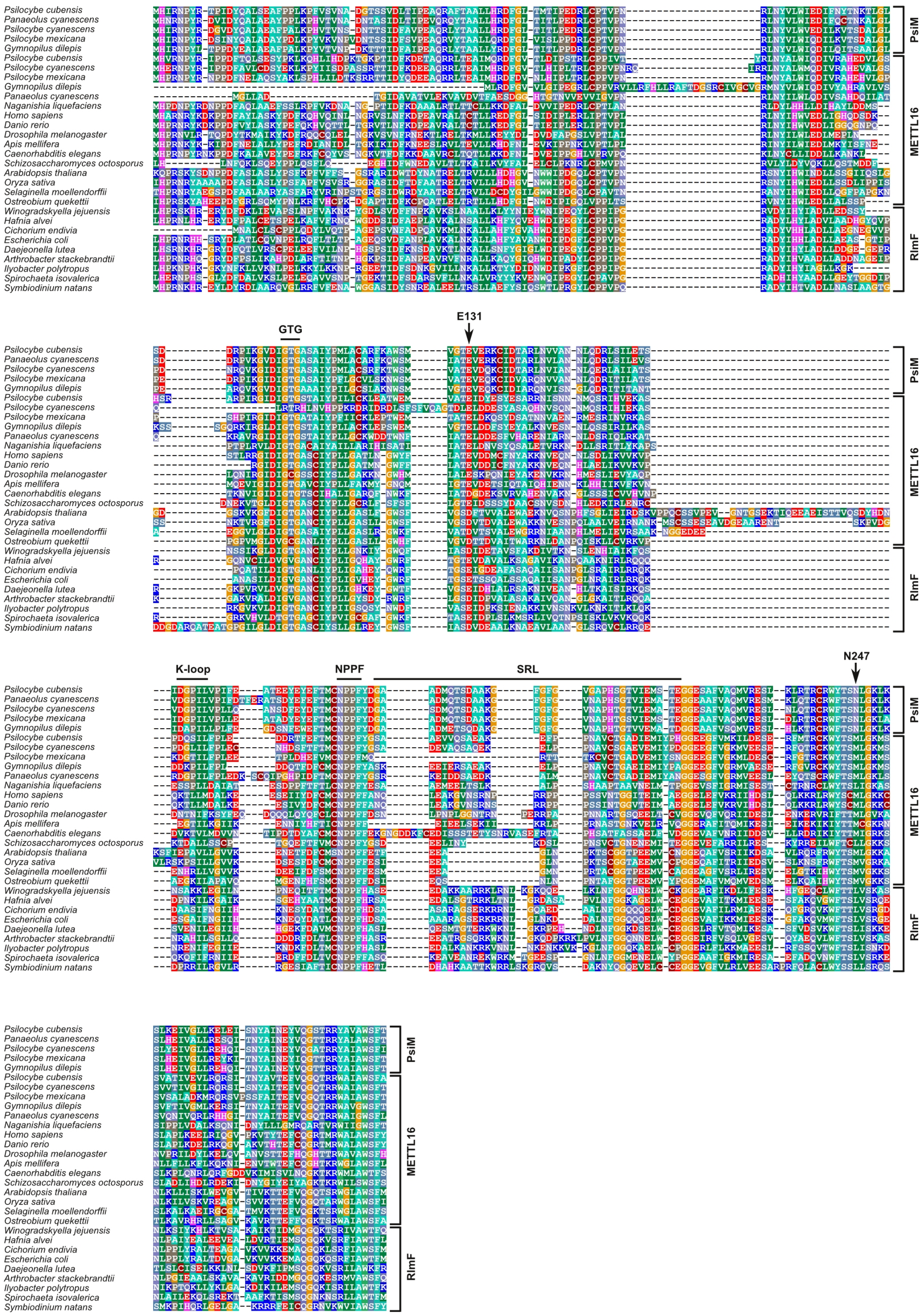

Annotated sequence alignment of the methyltransferase domains of PsiM, METTL16 and RlmF proteins from representative species.

Supplementary Fig. 5

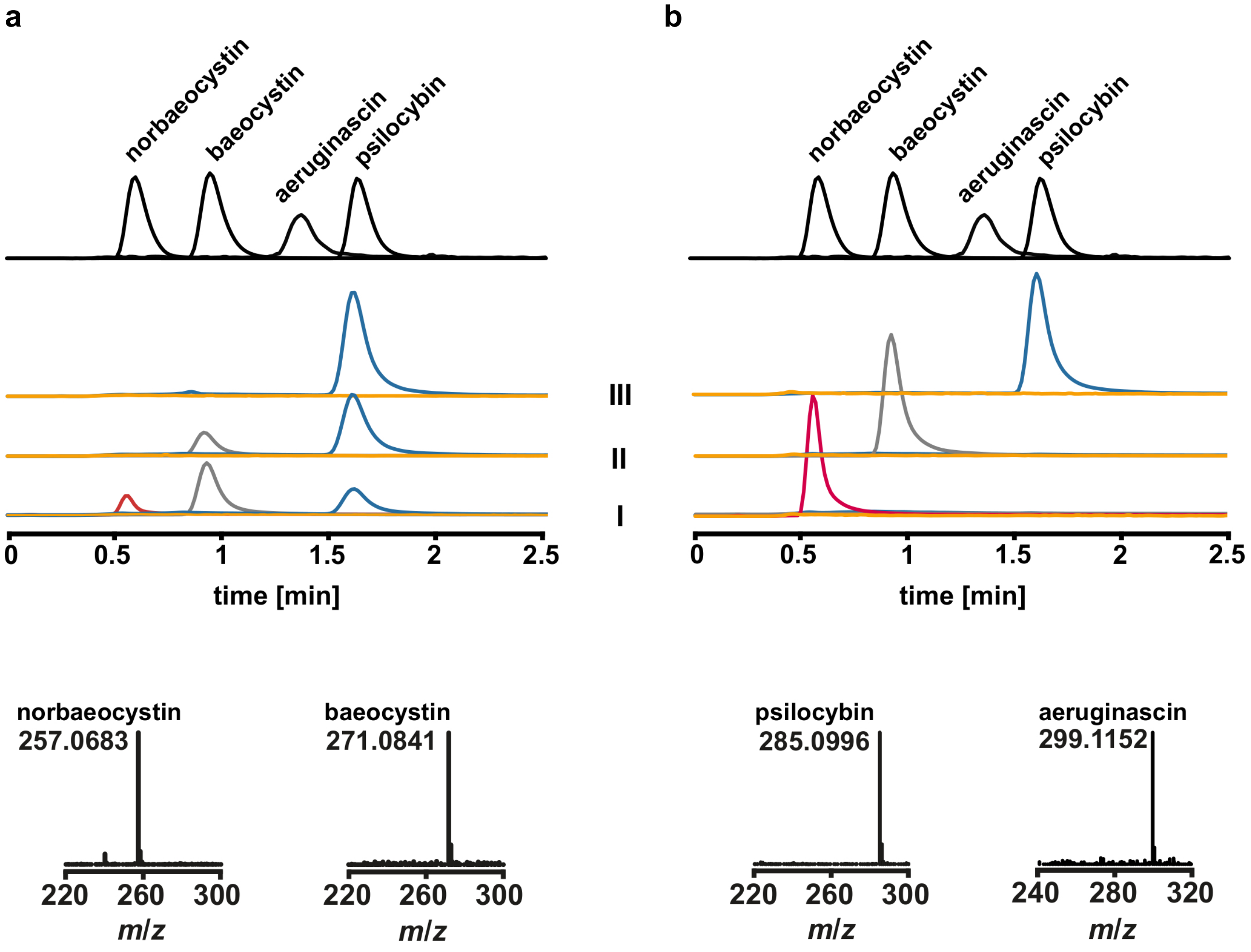

***In vitro* product formation assays with PsiM.**

a. Enzymatic reactions with norbaeocystin (trace I), baeocystin (II), and psilocybin (III). Shown are single ion chromatograms of norbaeocystin (red chromatogram, *m*/*z* 257 $[M+H]^+$), baeocystin (grey, *m*/*z* 271 $[M+H]^+$) and psilocybin (blue, *m*/*z* 285 $[M+H]^+$). Top panel: overlaid individual chromatograms of authentic standards. HR-ESIMS spectra (bottom) were recorded in positive mode and extracted from the chromatograms of the standards.

b. Negative controls with heat-inactivated enzyme.

Supplementary Fig. 6

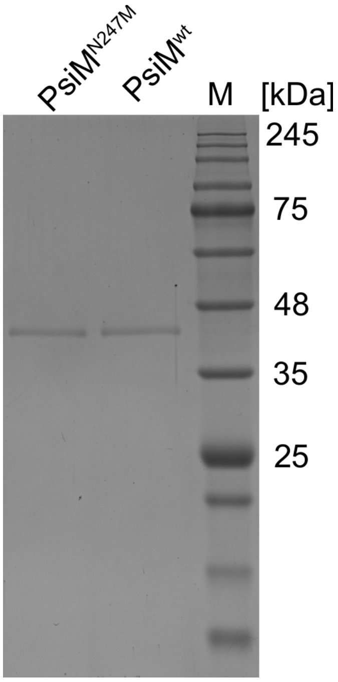

SDS-PAGE analysis of heterologously produced PsiM.

Coomassie-stained SDS-PAGE of the purified recombinant proteins used in enzymatic activity assays. M: molecular weight marker.

Supplementary Table 1

Data collection and structure refinement statistics. All datasets were collected from single crystals. Values in parentheses pertain to the highest resolution shell.

PDB entry	8PB3	8PB4	8PB5	8PB6	8PB7	8PB8	8QXQ
Ligands	SAH	SAH	Sinefungin	SAH	Sinefungin	SAH	SAH
	Norbaeocystin	Norbaeocystin	Norbaeocystin	Baeocystin	Baeocystin		Psilocybin
Lattice system	Monoclinic	Orthorhombic	Orthorhombic	Orthorhombic	Orthorhombic	Tetragonal	Orthorhombic
**Data collection**							
X-ray source	ESRF ID23-1	ESRF ID23-1	ESRF ID23-1	ESRF ID23-1	ESRF ID23-1	ESRF ID23-2	ESRF ID23-1
Wavelength (Å)	0.6888	0.6888	0.6888	0.6888	0.6888	0.8731	0.7293
Detector	DECTRIS EIGER2	DECTRIS EIGER2	DECTRIS EIGER2	DECTRIS EIGER2	DECTRIS EIGER2	DECTRIS	DECTRIS EIGER2
	X CdTe 16M	X CdTe 16M	X CdTe 16M	X CdTe 16M	X CdTe 16M	PILATUS3 X 2M	X CdTe 16M
Space group	$C2$	$P2_12_12_1$	$P2_12_12_1$	$P2_12_12_1$	$P2_12_12_1$	$P4_3$	$P2_12_12_1$
Unit cell dimensions							
a, *b*, *c* (Å)	166.0, 48.85, 115.5	49.23, 78.27, 83.77	49.21, 78.35, 83.80	49.23, 78.54, 83.73	49.37, 78.24, 83.81	62.20, 62.20, 154.8	49.34 78.52 83.93
α, β, γ (º)	90, 131.4, 90	90, 90, 90	90, 90, 90	90, 90, 90	90, 90, 90	90, 90, 90	90, 90, 90
V_m (Å^3/Da)	2.45	2.25	2.25	2.26	2.26	2.09	2.27
Solvent content (%)	49.8	45.4	45.4	45.5	45.5	41.1	45.8
Resolution range (Å) *	20.69 – 1.180	15.39 – 0.9100	12.92 – 0.8900	15.27 – 0.9300	15.41 – 0.9200	38.71 – 2.531	15.44 – 0.9400
	(1.222 – 1.180)	(0.9427 – 0.9100)	(0.9218 – 0.8900)	(0.9632 – 0.9300)	(0.9530 – 0.9200)	(2.622 – 2.531)	(0.9736 – 0.9400)
Total reflections	3162183 (320818)	3100048 (308778)	6649068 (662304)	2929157 (294033)	3021936 (302991)	117345 (10866)	5666662 (563837)
Unique reflections	225046 (19200)	230836 (20907)	247003 (23421)	217116 (19758)	224244 (21394)	19419 (1786)	211345 (20765)
Multiplicity	14.1 (14.5)	13.4 (13.6)	26.9 (27.1)	13.5 (13.7)	13.5 (13.6)	6.0 (6.0)	26.8 (26.9)
Completeness (%)	97.21 (84.57)	99.06 (91.50)	99.42 (95.79)	99.06 (91.74)	99.53 (96.22)	99.06 (91.68)	99.81 (99.16)
$\langle I/\sigma(I) \rangle$	8.25 (0.31)	10.37 (0.31)	13.39 (0.39)	11.39 (0.45)	10.88 (0.33)	5.38 (0.81)	13.36 (0.53)
Wilson *B* (Å^2)	16.70	11.02	11.33	10.43	11.20	45.23	11.17
R_{merge}	0.1241 (2.954)	0.08633 (3.04)	0.09517 (4.42)	0.08835 (2.682)	0.08247 (2.596)	0.3496 (2.197)	0.1082 (4.584)
R_{meas}	0.1289 (3.06)	0.08979 (3.16)	0.09700 (4.504)	0.09186 (2.785)	0.08574 (2.696)	0.3828 (2.402)	0.1103 (4.673)
R_{pim}	0.03473 (0.797)	0.02445 (0.857)	0.01863 (0.8593)	0.0249 (0.7457)	0.02327 (0.7213)	0.1543 (0.9646)	0.0212 (0.8985)
$CC_{1/2}$	0.996 (0.374)	0.997 (0.383)	0.999 (0.431)	0.999 (0.399)	0.999 (0.441)	0.977 (0.249)	0.998 (0.383)
CC^*	0.999 (0.738)	0.999 (0.744)	1 (0.776)	1 (0.755)	1 (0.782)	0.994 (0.631)	1 (0.745)
**Refinement**							
Nr. reflections							
used in refinement	221567 (19200)	228792 (20907)	245675 (23417)	215214 (19756)	223269 (21394)	19403 (1786)	211014 (20763)
in test set	3287 (275)	3439 (320)	3682 (353)	3233 (288)	3343 (308)	1050 (80)	3177 (317)
R_{work}	0.1827 (0.3767)	0.1443 (0.4057)	0.1393 (0.3862)	0.1381 (0.3542)	0.1462 (0.4068)	0.1973 (0.3052)	0.1365 (0.3541)
R_{free}	0.1955 (0.3629)	0.1558 (0.4173)	0.1514 (0.3904)	0.1524 (0.3596)	0.1612 (0.4083)	0.2513 (0.3758)	0.1493 (0.3648)
CC_{work}	0.969 (0.686)	0.976 (0.711)	0.974 (0.743)	0.974 (0.743)	0.972 (0.740)	0.956 (0.646)	0.976 (0.699)
CC_{free}	0.966 (0.694)	0.964 (0.692)	0.975 (0.732)	0.978 (0.745)	0.978 (0.730)	0.933 (0.513)	0.970 (0.695)
Nr. protein residues	620	320	320	320	320	622	320
Nr. non-hydrogen atoms							
total	5799	3253	3266	3262	3260	4998	3262
protein	4995	2748	2739	2745	2748	4892	2745
(co)substrate	86	60	88	62	72	52	64
other non-solvent	5	2	2	2	2	33	2
solvent	713	443	437	453	438	21	451
RMSD bond lengths (Å)	0.013	0.011	0.012	0.010	0.009	0.004	0.013
RMSD bond angles (º)	1.31	1.27	1.23	1.16	1.11	0.65	1.32
Ramachandran							
favoured (%)	98.21	98.74	98.74	99.06	98.43	98.04	98.43
allowed (%)	1.62	1.26	1.26	0.94	1.57	1.80	1.57
outliers (%)	0.16	0.00	0.00	0.00	0.00	0.16	0.00
Rotamer outliers (%)	0.73	0.65	0.33	1.30	0.98	1.85	0.98
Clash score	1.59	2.33	1.43	1.43	1.96	3.55	1.79
Average *B* (Å^2)							
overall	23.88	17.17	17.95	16.17	17.37	48.70	16.66
protein	22.98	15.34	16.15	14.34	15.72	48.65	14.61
ligands	19.50	13.92	11.59	12.70	11.08	62.24	15.66
solvent	30.50	28.87	30.60	27.66	28.74	38.08	29.31

* The significant digits of resolution ranges originate from reflection binning and do not represent resolution limit precision.

Supplementary Table 2: List of reagents

Reagent	Company	Catalogue Nr.
S-(5′-Adenosyl)-L-homocysteine (for activity assays)	Santa Cruz	sc-215826C
S-(5′-Adenosyl)-L-Methionin-*p*-toluolsulfonat	Sigma Aldrich	A2408-100MG
Sinefungin (for activity assays)	Medchemexpress	HY-101938
Imidazole	Carl Roth GmbH	x988.4
Tris	Carl Roth GmbH	A411.1
Sodium dihydrogen phosphate monohydrate	Carl Roth GmbH	K300.3
Di-Sodium hydrogen phosphate dihydrate	Carl Roth GmbH	4984.1
MES	Carl Roth GmbH	4256.2
Glycylglycine	Carl Roth GmbH	HN74.2
CAPSO	Carl Roth GmbH	5584.1
Sodium chloride	Carl Roth GmbH	3957.1
Methanol	VWR	20864.32
Formic acid	VWR	84865.26
BlueEye Prestained Protein Marker	Jena Bioscience	PS-104
L-Rhamnose	Carbolutions	CC20022
Trypton	Carl Roth GmbH	8953.4
Yeast extract	Carl Roth GmbH	2363.4
Polyethylene glycol (PEG) 8000	Carl Roth GmbH	0263.2
Isopropyl β-D-thiogalactopyranoside (IPTG)	Carl Roth GmbH	2316.4
Kanamycin	Carl Roth GmbH	T832.4
Glucose	Carl Roth GmbH	X997.2
Dithiothreitol (DTT)	Carl Roth GmbH	6908.3
Calcium chloride	Carl Roth GmbH	5239.1
Thrombin	Merck	T7326-1KU
Magnesium chloride	Carl Roth GmbH	KK36.3
Sinefungin (for crystallisation assays)	VWR	APOSBIS1400-1MG
S-(5′-Adenosyl)-L-homocysteine (for crystallisation assays)	Merck	A9384-10MG

Primers

oJF105	5‘-TATATAGCTAGCGAGAATCTTTATTTTCAGGGCATGCATATCAGAAATCC-3’
oJF47	5‘-TATATACTCGAGCTAGAAAAGAGAGCTGAG-3’
